# A comparative study of self-praise on English and Chinese social media: strategies, themes, and motivations

**DOI:** 10.3389/fpsyg.2023.1279853

**Published:** 2023-12-14

**Authors:** Hanwei Wu, Lehua Zhang, Shuai Ren

**Affiliations:** ^1^School of Foreign Studies, Hunan Normal University, Changsha, China; ^2^School of Foreign Studies, Hunan Applied Technology University, Changde, China; ^3^School of Politics and Public Administration, South China Normal University, Guangdong, China

**Keywords:** self-praise, social media, discourse analysis, cross-cultural comparison, psychological motivations

## Abstract

The act of self-praise, a novel and prevalent discourse pattern on social media across different cultures, is still understudied. As such, this study delves into self-praise from linguistic and psychological perspectives by examining and comparing the strategies, themes, and motivations of self-praise on English and Chinese social media. A discourse-based analysis of the data comprising 200 posts from Twitter (English) users and 200 posts from Weibo (Chinese) users revealed that Twitter users favored more unmodified explicit self-praise strategies, while Weibo users opted for more implicit self-praise strategies. Both groups employed explicit self-praise equally, but with different sub-strategies. The most prevalent themes were appearance, competence/performance, skills, virtues, and self-encouragement. Appearance was more dominant among Twitter users, whereas skills and self-encouragement were more popular among Weibo users. Both groups valued competence/performance and virtues, but with different virtues. Twitter users valued cherishing friendship, bravery, and maturity, whereas Weibo users stressed kindness, cherishing kinship, and thoughtfulness. Two semi-structured interviews conducted with six Twitter participants and six Weibo participants indicated that the motivations for online self-praise in both groups were psychological needs such as self-flattery, self-presentation, and self-encouragement, while commercial profit was a motivation only for Weibo participants. This study also discusses the social and cultural factors that may account for these differences and provides implications for future research on online self-praise.

## 1 Introduction

English and Chinese are two languages that have been widely studied in pragmatics, mainly because of the large population of their users and the fascinating and different cultures that underlie their politeness (Leech, 2007). Numerous studies have explored and compared various speech acts, especially those related to Brown and Levinson’s (1987) face theory such as gratitude, refusal, and requests, in English and Chinese (Zhu, 2017; Chang and Ren, 2020; House and Kádár, 2021; Li, 2022). However, the speech act of self-praise remains relatively understudied (Li and Wu, 2022; Xia and Jiang, 2022; Zhang and Aliya, 2022).

Self-praise is a type of self-presentation that involves disclosing positive information about oneself with the purpose of constructing a favorable self-image, which usually poses a threat to the hearer’s face to some extent (Ren and Guo, 2020). In the era of social media, self-praising content has become prevalent, which challenges the existing politeness theories. Therefore, investigating self-praise in online contexts may have pragmatic significance. Driven by this motivation, many studies have examined online self-praise in different contexts and languages and provided valuable insights into this politeness-deviant phenomenon (Dayter, 2014; Matley, 2018; Guo and Ren, 2020; Ren and Guo, 2020; Xia and Jiang, 2022).

However, except for several comparative studies (Ma et al., 2017; Chen and Lunt, 2021; Wan, and Yeung, 2022), few studies have directly compared online self-praise performed by English and Chinese speakers. Moreover, most of the existing literature has focused on the analysis of pragmatic strategies of self-praise employed by netizens and overlooked the themes of and motivations for performing self-praise online, which prevents from a more comprehensive understanding of this phenomenon (Dayter, 2014; Matley, 2018; Guo and Ren, 2020; Ren and Guo, 2020; Xia and Jiang, 2022). Therefore, to fill in these research gaps and provide references for online intercultural communication, this study aims to investigate and compare self-praise in terms of strategies, themes, and motivations in English and Chinese on social media.

## 2 Literature review

### 2.1 Theories in relation to self-praise

The existing literature on self-praise is scarce and scattered across different disciplines. This section reviews some of the relevant theories from sociopsychology and sociolinguistics that can shed light on the phenomenon of self-praise.

One of the sociopsychological perspectives that can be applied to online self-praise is Goffman’s (1959) self-presentation theory, which posits that people present themselves in different ways to construct an ideal identity for various purposes, such as obtaining job opportunities or influencing others’ impression (Speer, 2012). Self-presentation encompasses several psychological concepts, such as impression management (Pandey, 2022), self-concept (Strimbu and O'Connell, 2019), and self-esteem (Jang et al., 2018). Self-praise is a specific form of self-presentation that involves highlighting one’s positive attributes or achievements. Ellis et al. (2002) distinguish self-praise from other types of self-presentation, such as self-enhancement and ingratiation.

Another perspective that can provide more in-depth insights into online self-praise is politeness theory, which originates from sociolinguistics. Politeness theory deals with how people manage their face needs and avoid face-threatening acts in communication. Brown and Levinson (1987) propose one of the most influential models of politeness theory, which consists of three basic notions: face, face-threatening acts, and politeness strategies. Face refers to “the public self-image that every member wants to claim for himself,” which can be divided into positive face (the desire for one’s wants to be recognized, admitted, praised) and negative face (the hope for one’s unwillingness to be refuted, denied, criticized). Self-praise can be seen as a face-threatening act to hearers’ positive face, since it implies speakers’ superiority over others.

Leech’s (1983) politeness principle is another relevant theory from sociolinguistics, which is a continuation and revision of Grice’s (1975) cooperative principle. Leech (1983) argues that politeness plays a pivotal role in the establishment and maintenance of harmonious interpersonal relationships, which can be achieved by following six maxims: tact, generosity, approbation, modesty, agreement, and sympathy. The modesty maxim is most related to self-praise, as it states “minimize the expression of praise of self; maximize the expression of dispraise of self.” In other words, the modesty maxim encourages speakers to depreciate themselves and evaluate hearers positively. Self-praise clearly violates the modesty maxim in its nature.

The two sociolinguistic theories that focus on the maintenance of hearers’ face, but largely neglect the relational and interactional aspects of face, such as the maintenance of speakers’ face. To address this gap, Spencer-Oatey (2002) proposes rapport management theory, which accounts for the reciprocal politeness observed in daily communication. According to Spencer-Oatey (2002), communication should achieve a balance between the face needs of speakers and hearers. This implies that speakers should avoid being excessively modest or self-evaluating in a conversation. Self-praise, which involves a high degree of positive self-enhancement, seems to deviate from the essence of rapport management theory as well.

In conclusion, self-praise, as a new and prevalent discourse pattern in social media across the world, seems to contradict some core components of the existing politeness theories to some extent. Therefore, it is significant to explore self-praise in communication, especially in social media contexts.

### 2.2 Studies in relation to online self-praise

This study adopts the definition of self-praise proposed by Ren and Guo (2020), who regard it as a speech act that speakers use to display their valued possessions, such as their appearance, wealth, skills, and virtue. The existing research on self-praise can be divided into two main areas: self-praise in face-to-face communication and self-praise in online communication. The latter area, which is more pertinent to this study and has received growing interest in recent years, will be the main focus of this section. In particular, this section reviews the scarce literature on online self-praise, which is mostly conducted within pragmatics, and which either investigated the pragmatic strategies of a specific group in a single social media platform (Dayter, 2014; Guo and Ren, 2020; Rüdiger and Dayter, 2020) or explored those of the general users in one specific social media platform (Matley, 2018; Tobback, 2019; Ren and Guo, 2020; Xia and Jiang, 2022; Zhang and Aliya, 2022).

Concerning the self-praise strategies used by certain groups, Dayter (2014) conducted a pioneering study by examining how ballet students showcased their valued possessions on Twitter. She collected 1,000 posts and identified five self-praise strategies that the students employed to mitigate the potential negative effects of self-praise: providing a disclaimer, diverting the attention, making self-deprecating remarks, acknowledging hard work, and presenting it as a third-party complaint. She claimed that self-praise was a discursive practice to construct a ‘hero’ identity of a professional dancer. Rüdiger and Dayter (2020) later investigated another group: pick-up artists. By analyzing 38 texts, they identified three main types of self-praise strategies used by pick-up artists on forums: brag statements, proxy brags, and evidential brags. They also analyzed the reactions to self-praise and found that it was not challenged as it was part of pick-up artists’ interactional norms. Self-praise was also a way to construct the image of a successful seducer. Guo and Ren (2020) identified celebrities as another specific group that employed self-praise strategies on Weibo, a popular microblogging platform in China. They found three main categories of self-praise strategies after analyzing 300 posts: modified explicit self-praise, implicit self-praise, and explicit self-praise without modification. These studies showed that although they all tried to balance between self-promotion and politeness, different groups have different preferences for self-praise strategies. Ballet students and Chinese celebrities preferred indirect strategies (e.g., adding disclaimer, modified explicit self-praise), while pick-up artists liked direct strategies (e.g., brag statements).

For self-praise strategies employed by general users, Matley (2018) investigated how Instagram users performed self-praise and face work in their self-presentation when they posted photos that highlighted their achievements or qualities. He analyzed 200 posts and discovered that the hashtag #humblebrag was employed as an indirect and implicit self-praise strategy, which could be interpreted as a face-saving act that respected the audience’s negative face. Tobback (2019) examined self-praise on another online platform, LinkedIn, by comparing 90 LinkedIn summaries from French and US users. He revealed that both direct and indirect self-praise strategies were used on LinkedIn, but there were cultural differences in the degree of modification. Specifically, French users tended to use more indirect and softening strategies, while US users preferred more direct and strengthening strategies. In the Chinese context, Ren and Guo (2020) developed a detailed taxonomy of self-praise strategies used by Chinese netizens on Weibo, based on 300 posts. Their taxonomy consisted of three main categories: explicit self-praise without modification, modified explicit self-praise, and implicit self-praise, each with several sub-strategies. Applying this taxonomy, Zhang and Aliya (2022) explored the self-praise strategies on Xiaohongshu, a newly-emerged social media platform in China, using 300 posts. Xia and Jiang (2022) extended the research on online self-praise in China by examining another aspect besides strategies, namely, themes. They analyzed 623 posts on Weibo and found that there was a correlation between strategies and themes. This was a breakthrough compared with previous studies that only focused on self-praise strategies.

### 2.3 Research gaps and this study

The existing studies have made valuable contributions to the understanding of self-praise in social media, which is a face-enhancing but hearer-unfriendly discourse pattern that deviates from the conventional politeness theory. However, there are still some gaps in the literature that need to be addressed.

First, the classification of pragmatic strategies of self-praise in the existing literature is somewhat unclear and overlapping in dimension. For example, Ren and Guo (2020) included both “reference to hard work” and “praise from a third party” as subcategories of “modified explicit self-praise.” However, the former is related to “strategies,” while the latter seems more related to “themes” or “topics” rather than “strategies.” Therefore, this classification needs to be revised to be more dimensionally consistent. Second, previous studies have mainly focused on pragmatic strategies in social media, and few studies have systematically explored the topics or themes of self-praise. Moreover, there is a lack of empirical research on the motivations that drive users to perform self-praise or what users want to achieve by performing self-praise. Third, few studies have compared self-praise used by different users with different languages, which could enhance the understanding of different languages from a pragmatic perspective, and provide useful references for people who want to better assimilate into the cultures that different languages originate from. English and Chinese are the two languages with the most speakers in the world. Therefore, it is pragmatically significant to compare self-praise between English and Chinese.

To fill in the above-mentioned gaps in the literature, this study aims to achieve the following objectives: first, to investigate self-praise collected from Twitter and Weibo, two popular social network microblog platforms among English and Chinese speakers, respectively; second, to compare the strategies, themes, and motivations of self-praise performed by Twitter and Weibo users; and third, to explain the possible reasons for the differences. More specifically, this study is guided by the following research questions:

RQ1: What are the differences in strategies of self-praise between Twitter users and Weibo users?

RQ2: What are the differences in themes of self-praise between Twitter users and Weibo users?

RQ3: What are the differences in motivations for self-praise between Twitter users and Weibo users?

## 3 Materials and methods

### 3.1 Data collection and participants

This study employed data from Twitter and Weibo to investigate the strategies and themes of online self-praise. The search function and the hashtag feature of both platforms enabled the researchers to access public posts related to specific topics (Page, 2012; Sullivan, 2014). These features also helped the researchers to adopt an emic perspective and reduce their subjectivity in identifying self-praise posts. The keywords “self-praise,” “self-compliment” (without “-”), and “humblebrag” were used to search for posts on Twitter, and their Chinese equivalents “自夸,” “自我表扬,” and “自我赞赏” were used on Weibo. Following Matley (2018), 200 relevant posts from each platform were manually collected as the dataset. The 200 posts from Twitter came from 187 different users, while the 200 posts from Weibo came from 177 different users. To focus on ordinary users rather than celebrities, we selected the posts from users who had less than 200 followers, which was a stricter criterion than Ren and Guo (2020) who used 300 followers as the threshold.

To explore the motivations for online self-praise, we conducted online semi-structured interviews with 12 participants whose posts were in our dataset. The participants included 6 Twitter users (A-F; 3 males, 3 females; age: Mean = 25.3, *SD* = 4.72) and 6 Weibo users (1–6; 3 males, 3 females; age: Mean = 25.8, *SD* = 5.42). Specifically, we first contacted more than 30 users on each platform and received positive responses from 13 Twitter users and 8 Weibo users. Then we randomly selected 6 positive responders from each platform and interviewed them via video call on Instagram for Twitter users and on Wechat for Weibo users. The interview questions were: “why do you perform self-praise on Twitter? Can you give some reasons” for Twitter users, and “您为什么要在微博自夸呢? 能给出一些原因吗?” for Weibo users. With their consent, we recorded and transcribed the interviews that lasted about 30 min each. All participants signed the informed consent form. The Ethics Committee of Hunan Normal University approved this study.

### 3.2 Data coding and analysis

As the above description shows, the taxonomy in previous work is likely less clear. Therefore, we slightly modified the coding scheme developed by Ren and Guo (2020) to make it dimensionally clearer and suitable for our data (see Table 1). In the following explanation, we first provide English examples, followed by Chinese examples.

**Table 1:** Taxonomy of self-praise strategies.

Strategies	Sub-strategies
**Unmodified Explicit self-praise**	/
	Praise from a third party
	Disclaimer
**Modified explicit self-praise**	Change of praise focus
	Comparison between aspects of oneself
	Comparison between oneself and others
	Self-praise as question
**Implicit self-praise**	Self-praise as narration
	Self-praise as sharing

Unmodified explicit self-praise refers to praise of oneself without modified words to reduce the intended self-aggrandization. [e.g., I′ m so beautiful! (我太漂亮了!)]. Modified explicit self-praise refers to praise of oneself with modified words to attenuate the intended self-aggrandization, consisting of five sub-strategies. (1) Praise from a third party is defined as praise of oneself by quoting other people’s evaluation [e.g., My colleague said I was trustworthy. (我同事说我值得信赖。)]; (2) disclaimer is used to deny some responsibility for praise of oneself, often marked by a negation [e.g., I am not bragging, but my skin is quite good! (不是我自夸，我皮肤太好了!)]; (3) change of praise focus is operationalized as praising other people or things associated with digital users to imply a positive self-image [e.g., My dad is handsome, and I look just like him! (我爸好帅，我俩长得很像!)]; (4) comparison between aspects of oneself is achieved by comparing two aspects of digital users themselves, usually with self-deprecation to show humility [e.g., I′ m careless, but I′ m kind. (虽然我很马虎，但挺善良。)]; and (5) comparison between oneself and others is realized by comparing digital users themselves with others [e.g., I′ m better than most people. (我比多数人强。)].

Implicit self-praise is defined as the post in which the semantic meaning of the text and the hashtag self-praise are mismatched at a surface level, consisting of three sub-strategies. (1) Self-praise as question refers to the post in which questions are raised to highlight commendable qualities to hearers [e.g., Tell me if I am the most humorous person? (告诉我，我是不是最幽默的?)]; (2) self-praise as narration refers to the post in which digital users seemingly narrate the events or feelings, but their intention of self-praise is hidden in their narration [e.g., People can hate me but I would move on. (人们可以讨厌我，但我会继续前行。)]; and (3) self-praise as sharing refers to irrelevant words combined with photos or hyperlinks in which the praiseworthiness of digital users is implied [e.g., The sun is so shining today (a photo of blue sky is added). (今日阳光明媚。)]. As many posts referred to multiple strategies, we coded each strategy separately, and did the same for coding themes.

We followed the six steps of thematic analysis (Braun and Clarke, 2006) to analyze the self-praise posts using NVivo 11.0. The steps were: familiarization, initial coding, searching for themes, reviewing themes, defining themes, and reporting themes. The first and second authors coded the data for strategies, themes, and motivations independently. They used their own interpretations to code themes and motivations, as there was no prior literature to guide them. For instance, they coded the post “*I just want to show my followers that I’m a good person*” as “self-presentation” motivation. They initially generated different numbers of codes for each category on Twitter and Weibo (Twitter: 212 and 206 codes for strategies, 215 and 208 codes for themes, and 18 and 21 codes for motivations; Weibo: 231 and 224 codes for strategies, 210 and 213 codes for themes, and 28 and 25 codes for motivations). They discussed and resolved their discrepancies and reached an agreement on 209 codes for strategies, 213 codes for themes, and 19 codes for motivations on Twitter, and 227 codes for strategies, 211 codes for themes, and 27 codes for motivations on Weibo. They also agreed on most of the names for the codes. The main disagreement was on how to label a group of codes related to the themes of self-praise. These codes could be labeled as either “performance” or “competence,” depending on the perspective. They sought the opinion of the third author, who suggested using the combined term “performance/competence” for this group of codes.

We need to point out several issues. First, we did not modify grammatical errors or improper expressions in the posts and interviews to ensure the authenticity of the data. Second, to protect their privacy, we removed all identifiable information such as users’ nicknames and IDs (Georgakopoulou, 2017). Third, we used English tweets for our data, but not all tweeters are from English-speaking countries. Also, online self-praise may vary among native English speakers due to different cultural norms (e.g., America versus Britain). This may limit the validity of our findings somewhat. Fourth, we used SPSS 26.0 to perform quantitative analyses to determine the differences and similarities between the two groups with the alpha level at 0.05.

## 4 Results

### 4.1 Strategies of self-praise

To answer RQ1, we compared self-praise strategies using 200 posts from Twitter and Weibo. We coded the posts for three types of self-praise strategies: unmodified explicit, modified explicit, and implicit. We found that Weibo users employed more diverse strategies than Twitter users, as shown in Table 2.

**Table 2:** Frequency and percentage of self-praise strategies.

Strategies	Twitter users		Weibo users	
	Frequency	Percentage	Frequency	Percentage
**Unmodified explicit self-praise**	**129**	**61.72%**	**110**	**48.46%**
**Modified explicit self-praise**	**54**	**25.84%**	**65**	**28.63%**
Praise from a third party	6	2.87%	16	7.05%
Disclaimer	9	4.39%	21	9.25%
Change of praise focus	25	11.96%	26	11.45%
Comparison between aspects of oneself	3	1.44%	4	1.76%
Comparison between oneself and others	11	5.26%	0	0%
**Implicit self-praise**	**26**	**12.44%**	**52**	**22.91%**
Self-praise as question	4	1.91%	3	1.32%
Self-praise as narration	`19	9.09%	47	20.70%
Self-praise as sharing	3	1.44%	2	0.88%
**Total**	**209**	**100%**	**227**	**100%**

A Chi-square test revealed a significant difference between the two platforms in terms of self-praise strategies (Chi-square = 10.5, *df* = 2, *p* < 0.05). Further analysis showed that Twitter users used more unmodified explicit self-praise, while Weibo users used more modified explicit and implicit self-praise.

As some cells had an expected value that was 5 or less, we performed a Fisher’s exact test to examine the sub-strategies within each category and found significant differences in some of them. For modified explicit self-praise, Twitter users used less ‘praise from a third party’ (*p* < 0.05) and ‘disclaimer’ strategies than Weibo users (*p* < 0.05), but more ‘comparison between oneself and others’ strategies (*p* < 0.05). For implicit self-praise, Weibo users used more ‘self-praise as narration’ strategies than Twitter users (*p* < 0.05). The following examples illustrate these sub-strategies. The context of the posts is explained in square brackets.

#### 4.1.1 Unmodified explicit self-praise

Example 1 (Twitter users).
  I have great skin. Screw makeup and screw a filter [Followed by the user’s selfie.]

Example 2 (Weibo users).
  组装小能手上线!我不管!我最棒![附鞋柜组装前后的两张图片] [Translation: an excellent assembler goes online! Whatever! I’m the best! (Followed by two photos of the shoe cabinet before and after assembly)].

### 4.2 Modified explicit self-praise

#### 4.2.1 Praise from a third party

Example 3 (Twitter).
  Everyone: Johnny, why are you so popular? Me: Because I love everyone!

Example 4 (Weibo).
  同学都说我跟一个代课老师长得像，话说她确实挺好看的。(Translation: My classmates say I look like a substitute teacher, and she is really pretty).

#### 4.2.2 Disclaimer

Example 5 (Twitter).
  I never say this, but I’m so proud of myself. Just reviewed my morning presentation and am so proud of how far I have come.

Example 6 (Weibo).
  不是自夸，但是我觉得我看男人的眼光真挺不错的。(Translation: I’m not bragging, but I think I have a pretty good eye for men).

#### 4.2.3 Comparison between oneself and others

Example 7 (Twitter).
  I’m honestly dying to show you the painting. I once said Doc Culber was the best one yet, I dare to say we have a new one.

### 4.3 Implicit self-praise

#### 4.3.1 Self-praise As narration

Example 8 (Twitter).
  I have completed the task I’ve been avoiding.

Example 9 (Weibo).
  今天一大爷的车，卡在柱子中间动不了了。我赶快去帮忙最后安全倒回来了。(Translation: Today an old man’s car got stuck in the middle of two pillars and could not move. I quickly went to help and finally backed it up safely).

### 4.4 Themes of self-praise

To address RQ2, we analyzed the themes of self-praise on Twitter and Weibo, using 200 posts from each platform, respectively. We coded the posts for five themes: appearance, performance/competence, skills, virtues, and self-encouragement. We found that the two platforms had similar numbers of themes, but different distributions, as shown in Table 3.

**Table 3:** Frequency and percentage of themes about self-praise.

Themes	Twitter users			Weibo users	
	Frequency	Percentage		Frequency	Percentage
**Appearance**	**62**	**29.11%**	**Appearance**	**35**	**16.59%**
**Performance/Competence**	**41**	**19.25%**	**Performance/Competence**	**40**	**18.96%**
**Skills**	**50**	**23.47%**	**Skills**	**64**	**30.33%**
Food making	29	13.62%	Food making	51	24.17%
Photographing	13	6.10%	Photographing	8	3.79%
Painting	8	3.76%	Painting	5	2.37%
**Virtues**	**48**	**22.54%**	**Virtues**	**38**	**18.01%**
Cherishing friendship	16	7.51%	Kindness	18	8.53%
Bravery	12	5.63%	Cherishing kinship	11	5.21%
Maturity	10	4.70%	Thoughtfulness	9	4.27%
**Self-encouragement**	**6**	**2.82%**	**Self-encouragement**	**16**	**7.58%**
**Others**	**6**	**2.82%**	**Others**	**18**	**8.53%**
**Total**	**213**	**100%**	**Total**	**211**	**100%**

A Chi-square test revealed a significant difference between the two platforms in terms of self-praise themes (Chi-square = 14.5, *df* = 4, *p* < 0.05). Further analysis indicated that Twitter users used more appearance-related self-praise, while Weibo users used more skills-related and self-encouragement-related self-praise.

Using a Fisher’s exact test, we also examined the sub-themes within each theme and found significant differences in some of them. For skills, Weibo users used more food-making-related self-praise than Twitter users (*p* < 0.05). For virtues, the two platforms had different sub-themes, such as cherishing friendship, bravery, and maturity on Twitter, and kindness, cherishing kinship, and thoughtfulness on Weibo. The following examples illustrate these sub-themes. The context of the posts is explained in square brackets.

### 4.5 Appearance

Appearance in our study refers to the way people look like. In our corpus, self-praise for good appearance was largely involved with various expressions and generally with selfies.

Example 10 (Twitter).
  I’m obsessed with myself and how far I’ve come as a person! I’m the fucking shit!!! [Followed by the user’s selfie.]

Example 11 (Weibo).
  这张不记得及时拍的, 但是觉得满漂亮。[附用户自拍] (Translation: I do not remember when I took this photo, but I think it’s very beautiful. [Followed by the user’s selfie.])

### 4.6 Performance/competence

In this study, performance refers to how successful people are or how well they do something, which indicates their competence. In our corpus, performance/competence can be roughly divided into three categories: performance/competence in dealing with issues in daily life, study, and work. Due to the ambiguity of many posts about performance/competence, we did not offer any subthemes.

Example 12 (Twitter).
  Cannot believe tomorrow is my last ever day on clinical placement as a student physiotherapist so proud of everything I’ve achieved so far.

Example 13 (Weibo).
  今日自夸:PhD期间应当有的重要训练:快速理解他人想法的能力。(Translation: Today’s self-praise: An important training that should be done during doctoral studies: the ability to quickly understand other people’s ideas).

### 4.7 Skills

In this study, skills refer to a type of work or activity that requires specialized knowledge or expertise. In our corpus, the three most common skills were culinary arts, photography, and painting among both Twitter and Weibo users.

#### 4.7.1 Food making

Example 14 (Twitter).
  After successfully preparing sambhar, dosa & coconut chutney that too from scratch which includes preparing sambhar masala & dosa batter, I now declare myself a great chef.

Example 15 (Weibo).
  自己做的，虽然不如外面的卖相好，但是自己做的干净，味道也不错的说!嘿嘿，让我自夸一下自己哈。(Translation: It’s homemade. It may not look as nice as the ones sold outside, but it’s clean and delicious. Hehe, let me brag a little about myself).

### 4.8 Virtues

In this study, virtues refer to moral or ethical qualities. Twitter and Weibo users showed significant differences in their emphasis on specific virtues. For Twitter users, the three most prevalent virtues were loyalty to friends, courage, and maturity. For Weibo users, they were kindness, respect for family, and thoughtfulness.

#### 4.8.1 Cherishing friendship

Example 16 (Twitter).
  I’m sweet and treat my partner well if I really like you. I’m the sweetest guy you’ll ever meet.

#### 4.8.2 Kindness

Example 17 (Weibo).
  13号线上，隔壁小情侣起身下车时，余光撇到座位上有个黑色物体，调经反射边叫他们边捡起了手机~啊 ~ 我真是个好宝宝，一个爱疯都不能动摇我。(Translation: On the subway Line 13, I caught a glimpse of a black thing on the seat when the young couple beside me stood up to leave. I instinctively shouted to them and picked up the phone. Ah, I’m such a good kid, even an iPhone cannot tempt me).

### 4.9 Self-encouragement

In this study, self-encouragement refers to how people motivate, encourage, uplift, and comfort themselves to overcome difficulties in their life. In our corpus, we found that people, especially Weibo users, often achieved self-motivation by performing self-praise.

Example 18 (Twitter).
  Little encouragement and support for all who might need it today. Keep on surfing, you are doing great!

Example 19 (Weibo).
  学会适可而止，看不完的电影可以暂停，咽不下去的食物可以不吃，人活一世，不要过于苛责自己。(Translation: Learn to stop when appropriate, pause the movie you cannot finish, do not eat the food you cannot swallow, live your life, do not be too hard on yourself).

### 4.10 Motivations for self-praise

To address RQ3, we conducted semi-structured interviews with 6 Twitter and 6 Weibo users. From the analysis of the qualitative data, we identified two themes related to implicit self-praise, namely, psychological needs and commercial profit (see Table 4).

**Table 4:** Motivations for self-praise in online contexts.

Themes	Subthemes
	Self-flattery
**Psychological needs** (T, W)	Self-presentation
	Self-encouragement
**Commercial profit** (W)	

T, the subthemes found in Twitter users; W, the subthemes found in Weibo users.

### 4.11 Psychological needs

Psychological needs comprised three subthemes: self-flattery, self-presentation, and self-encouragement. Self-flattery involves excessive pride in oneself. In our study, self-flattery, found in both groups, is one’s desire to receive praise from others to increase their sense of superiority. Superiority can derive from appearance, competence, skills, and so on. For instance, participant D and participant 3 stated:

You know, Twitter is where I show off all the amazing things I’ve done in different areas. I have a lot to be proud of, because I’ve achieved things that most people can’t even dream of. I think people need to see how awesome I am and learn from me. (Participant D—Interview for Twitter users)

I don’t want to show off on WeChat. You know, ironically, the ones who don’t want you to do well are always your acquaintances. But on Weibo, I don’t have any acquaintances. Flaunting my achievements can make netizens think I’m a smart guy. Honestly, praising myself on Weibo can satisfy my vanity. (Participant 3—Interview for Weibo users)

In this study, self-presentation refers to how people portray themselves, including their physical appearance and their impressions of personality, competence, and so on. The difference between self-presentation and self-flattery is that the latter implies superiority over others. Both Twitter and Weibo users mentioned their hope to establish a positive image through performing self-praise. For instance, participant B and Participant 6 said:

I enjoy boasting on Twitter. I just want to show my followers that I’m a good person, even if I don’t have many followers. (Participant B—Interview for Twitter users)

I want to present myself as a confident and successful person on Weibo, so I often post about my achievements and accomplishments. I think this way, I can impress others and gain their admiration and respect. I also hope that by sharing my positive experiences, I can inspire and motivate others to pursue their goals. (Participant 6—Interview for Weibo users)

Self-encouragement refers to how users employed self-praise to encourage, console, or uplift themselves, so that they could overcome difficulties, mitigate negative emotions, or forgive themselves for their regrets, mediocrity, and so on. Self-encouragement was found in both groups, as indicated in the following excerpt:

When I feel low, I try to boost my confidence by telling myself that I’ve done a great job. Twitter gives me a space where I can express my weaknesses and recharge myself because nobody knows me. (Participant E—Interview for Twitter users)

I often face setbacks in my life that make me feel frustrated, so I use self-praise to raise myself up. I share my strengths and achievements on Weibo, and tell myself how much progress I have made. I believe this way, I can increase my confidence, and deal with the difficulties more optimistically. (Participant 2—Interview for Weibo users)

### 4.12 Commercial profit

Self-praise for commercial profit was only reported by Weibo participants. Traditionally, commercial profit derives from the sale of commodities at prices above those paid by merchants. However, Weibo users can gain commercial profit by posting advertisements. For instance, participant 6 mentioned:

Many online users get rich overnight. I don’t expect to be like them. But it would be nice if I could earn some extra money. Obviously, if you want to attract advertisers, you need a lot of followers. Showing off your skills and good looks is probably the best way to get more followers. (Participant 6—Interview for Weibo users)

I often praise myself for being a good cook and a food lover on Weibo. I post pictures and videos of the dishes I make or the restaurants I visit. I also write some reviews and tips for cooking or eating. I hope to attract more followers and then get paid by some food brands or restaurants to endorse their products or services. This way, I can make some extra income and also showcase my skills and taste. (Participant 1—Interview for Weibo users)

## Discussion

In this study, we investigated and compared self-praise strategies, themes, and motivations in English and Chinese online contexts. It is noteworthy that we only incorporated posts containing “self-praise” and its synonyms in hashtags. Therefore, the proportion and category of self-praise strategies and themes may differ in self-praise posts without these hashtags. This problem is almost inherent in empirical dataset-based research in a computer-mediated communication environment.

In relation to RQ1, our first finding was that Twitter users used unmodified explicit self-praise more frequently than Weibo users, which is in line with previous literature on self-positivity in the field of culture and psychology (Xie and Teo, 2020; Lou and Li, 2022; Salvador et al., 2022). For instance, Salvador et al. (2022) compared self-enhancement and self-criticism in Western (American) and Eastern (Chinese) samples. They confirmed that the Western sample exhibited self-enhancement bias, while the Eastern sample showed self-criticism bias. Therefore, cultural differences may also explain why Twitter users preferred unmodified explicit self-praise more. Moreover, Western people, who usually come from low-context or individualistic cultures, tend to communicate directly, while Eastern people, who usually come from high-context or collectivistic cultures, tend to communicate indirectly (Velez-Calle et al., 2021; Yang et al., 2021; Tabata and Vrij, 2023). We assume that this difference also affects the way Twitter and Weibo users employ self-praise.

Secondly, we found that that both groups utilized modified explicit self-praise with a similar frequency, but they exhibited different preferences for the sub-strategies they employed. Specifically, Weibo users favored the use of praise from a third party and disclaimer as modes of expressing self-praise, whereas Twitter users resorted to more comparisons between oneself and others. Praise from a third-party entail quoting or mentioning positive evaluations from others. This sub-strategy can render self-praise more objective and less face-threatening, as it enlarges the context and elicits more responses from the interlocutors (Guo and Ren, 2020; Itakura, 2022). Disclaimer is another sub-strategy that entails displaying modesty or disavowing self-praise. This sub-strategy can also diminish the threat to face by distancing oneself from self-praise and avoiding arrogance (Ren and Guo, 2020; Xia and Jiang, 2022). Hence, Weibo users’ proclivity for these sub-strategies reflects their adherence to politeness theories and Chinese traditional culture that value modesty and humility (Ren and Guo, 2021; Shi et al., 2021; Lin and Chen, 2022). In contrast, Twitter users’ frequent use of comparison between oneself and others is a sub-strategy that entails accentuating one’s achievements or qualities in relation to others. This sub-strategy implies a sense of superiority and competitiveness, which may be influenced by western culture’s great value on competition. Typically, Western beliefs, influenced by Protestantism, emphasize competition over cooperation (Dagnino and Minà, 2021), while Eastern beliefs, influenced by Confucianism, emphasize cooperation over competition (Minà and Dagnino, 2021).

Our third finding was that Weibo users and Twitter users differed in their frequency of using implicit self-praise. Weibo users employed this strategy more frequently than Twitter users. This finding is in line with the empirical study of Cai et al. (2016), which suggests that Eastern samples (China) tend to downplay the positivity of the self more directly than Western samples (America) due to prohibitive cultural pressures. However, Eastern samples also tend to indirectly highlight the positivity of the self through their humility or situationally triggered behavior. Thus, we attribute Weibo users’ higher frequency of using implicit self-praise to these cultural differences. Among the individual strategies of implicit self-praise, Weibo users favored self-praise as a narration more than Twitter users. This is consistent with the findings of Xia and Jiang (2022) that Weibo users often provided evidence through narration when they performed self-praise. It implies that the modesty maxim still operates and exerts a greater influence on the social interaction of Chinese speakers than English speakers.

With respect to RQ2, our first finding was that both Twitter and Weibo users employed appearance as a most repeated theme when they performed self-praise, but Twitter users emphasized appearance more. Previous studies observed that self-praise of appearance accompanied by selfies was prevalent among both groups, but they did not compare the frequency of these two groups (Ma et al., 2017; Ren and Guo, 2020). The preference for appearance-related self-praise among both groups may be attributed to the fact that people from different cultures share the same strong desire to construct and display their positive social image and ideal selves, particularly in online contexts, although the extent of their desire may be nuanced (Burnell et al., 2021; van Oosten et al., 2023). Furthermore, as indicated earlier, the modesty maxim exerts more constraints on Eastern people. Self-praise on appearance may entail higher risks of threatening the face of hearers than other forms of self-praise, since self-praise on genetically determined appearance could be interpreted as discrimination against hearers who are not good-looking (Spiegel, 2023). Thus, Weibo users may be less inclined to perform appearance-related self-praise.

Another of our findings was that both groups gave equal importance to performance/competence as a theme when they performed self-praise. This theme was related to various aspects of their lives, such as academic performance, mundane life, and career. For example, they praised themselves for getting good grades, completing tasks, or achieving goals. This finding is not surprising, as performance/competence has been long recognized as one of the most valuable attributes in both cultures. Western culture’s emphasis on competence/performance can be traced back to Greek philosophy, in which competence and performance were defined as the ability and the action of achieving excellence and their relationship was explained in Aristotelian tradition (Trültzsch-Wijnen, 2020). Eastern people’s value on competence/performance is greatly influenced by Confucianism. Confucius stressed the salience of competence/performance many times in his lifetime. In his philosophy, competence/performance is a comprehensive concept that involves erudition and versatility, or the knowledge and the skill of applying it in different situations (Brown, 2021). The significance of competence/performance in both philosophical origins has a lasting impact on contemporary West and East. Competence/performance’s close associations with career success, perceived by ordinary people and demonstrated by researchers, may also make it one of the archetypal constructs of positive self-presentation (Elsey et al., 2022; Zhang et al., 2022; Peltokorpi, 2023).

We further discovered that Weibo and Twitter users utilized three skills as themes for their self-praise on social media. These skills were food making, photographing, and painting. Food making was the most preferred skill by Weibo users compared to Twitter users. The common use of these skills in both groups could be explained by their accessibility to ordinary people who can readily practice them in their daily lives, but the additional emphasis on food making by Weibo users might have underlying reasons associated with cultural and social factors. Food making or cooking, as a crucial component of domestic labor, could be viewed as the manifestation of housework (Guan and Zuo, 2021). The traditional gender norm that stipulates males as the breadwinner and females as the homemaker has been altered in China’s labor market due to the rising participation of women in the workforce, but it has remained intact in the domestic sphere where women still shoulder most of the domestic duties (Xu, 2021; Zhang and Xu, 2022). Consequently, females who exhibit their cooking skills online may aim to construct a positive image of a (potential) good wife who can balance work and family. Food making is also valued by Chinese males. Previous research indicated that Chinese urban husbands performed more domestic labor than their rural counterparts, implying a regional decrease of gender disparities in housework due to a socio-economic transition (de Bruin and Liu, 2020). Therefore, we hypothesize that many Chinese males also have a strong motivation to present their cooking skills on social media to portray themselves as a (potential) good husband who can share the domestic burden with their partners.

Additionally, we discovered that Twitter and Weibo users placed the same emphasis on virtues, but they differed in their focus on specific virtues that they employed to praise themselves on social media. Twitter users valued cherishing friendship, bravery, and maturity, whereas Weibo users stressed kindness, cherishing kinship, and thoughtfulness. Previous studies have indicated that westerners have a larger friendship network and derive more well-being from it than easterners, suggesting that westerners’ value of friendship is probably culturally rooted (Li and Cheng, 2015; Hongladarom and Joaquin, 2021). Expressing bravery and maturity are consistent with Western culture that is imbued with individualism, which emphasizes personal achievement and autonomy. Virtues in the contemporary East are profoundly influenced by their culture, history, and philosophy (Fatehi et al., 2020). The three virtues highlighted by Weibo users are all within the scope of Ren (benevolence), one of the basic notions of Confucian virtue ethics that guides moral conduct and interpersonal relationships. Confucius’s Ren has its ethical sequence: to love relatives first, which corresponds to cherishing kinship, and to love others afterward, which matches kindness (Hu et al., 2021; Lee, 2022). Thoughtfulness is also reflected by Ren, as the Confucian maxim states: “treat others as you hope they will treat you” (Jia, 2022), which implies reciprocity and empathy.

Interestingly, we also discovered that Weibo users showed a significantly higher frequency of posting content related to self-encouragement than Twitter users, which may have its social basis. Recently, the word “involution” has become extremely popular in Chinese-language social media as a complaint of hard life experienced by individuals in the contemporary era (Forges, 2022). Involution in the Chinese context refers to the situation where people make extra efforts to compete for limited resources, resulting in a decline in the “return on investment” of individuals. It could be seen as the “inflation of effort” that does not lead to proportional rewards or benefits. Involution has been demonstrated in many fields in China such as education and employment, widely affecting Chinese citizens (Dou et al., 2022; Yu et al., 2022). We assume that Weibo, due to its anonymity, is ideal for Chinese netizens facing involution to rejuvenate themselves through self-encouragement. In other words, self-encouragement becomes a form of self-praise that enhances Chinese netizens’ confidence and motivation so that they are empowered to cope with the societal pressures.

With respect to RQ3, we found that both Twitter and Weibo participants performed self-praise out of three psychological needs: self-flattery, self-presentation, and self-encouragement. These needs reflect different purposes and motivations for engaging in self-praise online. Previous literature has corroborated that netizens’ online self-praise was mainly for presenting the best possible self to others (Strimbu and O'Connell, 2019; Zheng et al., 2020; Kawamoto, 2021), which was also confirmed by our study. This type of self-praise, which we term self-presentation, aims to create a positive impression and gain social approval. However, our study, to our best knowledge, is the first one to empirically find that performing self-praise online is also possible for expressing superiority over others and for consoling and uplifting oneself. The former type of self-praise, which we term self-flattery, contradicts the modesty maxim in nature, since it threatens the hearers’ face by boasting one’s own achievements or qualities. The anonymity of these two platforms may facilitate self-flattery to occur as self-flatterers do not need to maintain hearers’ face when the intention of social interaction is lacking. The latter type of self-praise, which we term self-encouragement, is more prevalent among Weibo users than Twitter users. As discussed above, self-encouragement is the result of catharsis, which means releasing negative emotions and coping with stress by affirming one’s abilities and achievements.

Another noteworthy finding of our study is that performing self-praise for commercial profit was only observed in Weibo participants, which may be closely related to the prosperity of fan economy in China (Xu, 2023; Yang, 2023). Fan economy refers to the phenomenon where fans support their idols by purchasing their products or services. In recent years, many celebrities who used to be ordinary people with a huge number of fans have become millionaires through e-commerce livestreaming (Si, 2021), a form of online marketing that involves live video and interaction with potential customers. As a result, to benefit from this flourishing industry, an increasing number of ordinary people may be motivated to perform online self-praise for constructing a positive image to attract more fans. For instance, they may post content that showcases their talents, skills, or achievements. Naturally, these “would-be” celebrities share the same motivation for self-praise, namely, commercial profit, with those real celebrities (Guo and Ren, 2020), who also use self-praise as a means of promoting themselves and their products or services. Hence, self-praise for commercial profit is a type of self-praise that aims to generate income and gain popularity.

## 5 Conclusion

This study examined and contrasted how Twitter (English) and Weibo (Chinese) users perform self-praise and explored the potential factors behind the cultural differences from a linguistic and psychological perspectives. Regarding strategies, Twitter users favored more unmodified explicit self-praise, while Weibo users opted for more implicit self-praise. Both groups used explicit self-praise equally, but with different sub-strategies. Concerning themes, appearance, competence/performance, skills, virtues, and self-encouragement were most common. Appearance was more prevalent among Twitter users, and skills and self-encouragement were more popular among Weibo users. Both groups valued competence/performance and virtues, albeit with different virtues. In terms of motivations, psychological needs and commercial profit were identified. Psychological needs that included self-flattery, self-presentation, and self-encouragement were reported in both groups. Commercial profit was only observed in Weibo participants.

This study makes several contributions to the existing literature. Theoretically, this study is the first one, to the best of our knowledge, that adopts both linguistic and psychological perspectives to compare and contrast online self-praise behavior of English and Chinese speakers in terms of strategies, themes, and motivations. Practically, this study provides a deeper insight into the cross-cultural differences and psychological factors that influence online self-praise of the two groups across the three aspects, which has implications for intercultural communication.

However, this study also has some limitations. First, the sample sizes of posts and participants in this study were relatively small, which may affect the generalizability of the findings to a larger population. Therefore, future research should use larger and more representative samples to validate the results. Second, the findings may be subject to some bias, as different social media platforms may attract different types of posts and users. For example, in North America, people who want to showcase their culinary skills may prefer Instagram or TikTok over Twitter. Thus, future research should include more social media platforms (e.g., TikTok) to conduct comparative studies. Third, the language background of the Twitter users in this study may have an impact on their online self-praise behavior, as they may not all be native English speakers. This may affect the validity of our conclusions. In fact, even among native English speakers, there may be variations in online self-praise due to different cultural norms (e.g., America versus Britain). Therefore, future research should control for the language background by focusing on one specific English-speaking country. Fourth, we relied on interviews to explore the motivations for online self-praise, which were based on the participants’ self-reported psychological needs. However, these needs may not reflect their actual motivations, as they may be influenced by social desirability or other factors. Future research should use more objective and reliable methods to measure the motivations for online self-praise, such as experiments or observations (Derakhshan et al., 2023). Fifth, we did not investigate the possible relationships between self-praise strategies, themes, and motivations, as they were coded independently. Hence, a more comprehensive study that explores the links between these three aspects is recommended for future research to gain a deeper understanding of online self-praise behavior of the two groups.

## Data availability statement

The raw data supporting the conclusions of this article will be made available by the authors, without undue reservation.

## Ethics statement

The studies involving humans were approved by the Ethics Committee of Hunan Normal University. The studies were conducted in accordance with the local legislation and institutional requirements. The participants provided their written informed consent to participate in this study.

## Author contributions

HW: Conceptualization, Data curation, Formal analysis, Investigation, Methodology, Resources, Software, Supervision, Validation, Visualization, Writing – original draft, Writing – review & editing. LZ: Funding acquisition, Investigation, Methodology, Supervision, Writing – original draft, Writing – review & editing. SR: Data curation, Investigation, Supervision, Validation, Visualization, Writing – original draft.
